# Can NGOs regulate medicines markets? Social enterprise in wholesaling, and access to essential medicines

**DOI:** 10.1186/1744-8603-7-4

**Published:** 2011-02-28

**Authors:** Maureen Mackintosh, Sudip Chaudhuri, Phares GM Mujinja

**Affiliations:** 1 Department of Economics Faculty of Social Sciences The Open University, Walton Hall, Milton Keynes MK7 6AA, UK; 2 Indian Institute of Management Calcutta, Joka, Kolkata 700 104, India; 3 Muhimbili University of Health and Allied Sciences, PO Box 65015 Dar es Salaam, Tanzania

## Abstract

**Background:**

Citizens of high income countries rely on highly regulated medicines markets. However low income countries' impoverished populations generally struggle for access to essential medicines through out-of-pocket purchase on poorly regulated markets; results include ill health, drug resistance and further impoverishment. While the role of health facilities owned by non-governmental organisations (NGOs) in low income countries is well documented, national and international wholesaling of essential medicines by NGOs is largely unstudied. This article describes and assesses the activity of NGOs and social enterprise in essential medicines wholesaling.

**Methods:**

The article is based on a set of interviews conducted in 2006-8 with trading NGOs and social enterprises operating in Europe, India and Tanzania. The analysis applies socio-legal and economic perspectives on social enterprise and market regulation.

**Results:**

Trading NGOs can resist the perverse incentives inherent in medicines wholesaling and improve access to essential medicines; they can also, in definable circumstances, exercise a broader regulatory influence over their markets by influencing the behaviour of competitors. We explore reasons for success and failure of social enterprise in essential medicines wholesaling, including commercial manufacturers' market response; social enterprise traders' own market strategies; and patterns of market advantage, market segmentation and subsidy generated by donors.

**Conclusions:**

We conclude that, in the absence of effective governmental activity and regulation, social enterprise wholesaling can improve access to good quality essential medicines. This role should be valued and where appropriate supported in international health policy design. NGO regulatory impact can complement but should not replace state action.

## Introduction

The aims of this article are the following. We first aim to document the importance, for access to medicines in low income contexts, of the largely unresearched role of social enterprise in essential medicines wholesaling, drawing on a unique dataset of interviews undertaken in Europe, India and Tanzania. Second, we seek to explain the extent and limits of the market impact of this social enterprise wholesaling by using economic and socio-legal theory and our interview evidence to sketch an analytical understanding of the scope for social enterprise to be market-regulating. In developing this argument, we identify benefits that can flow from social enterprise trading; limitations placed on social enterprise success by commercial competition; and some conditions for the emergence of a distinct 'social market' segment of medicines markets where social enterprise can effectively shape the terms of exchange to the benefit of low income consumers. We conclude that a better understanding of the role of social enterprise in the problematic but socially important market for essential medicines, should be incorporated into health and development research and policy.

## Background

Most people in Africa and India lack regular access to safe essential medicines[1]. India has a highly developed pharmaceutical industry, yet appropriate reliable medicines do not reach most low income people in India nor in African countries to which India exports medicines[2]. Instead, these populations experience substandard medicines, inappropriate and incomplete treatments, excess ill health and mortality, drug-resistant disease, exclusion from treatment, and further impoverishment when they struggle to pay [1,3-6].

These outcomes result from extreme poverty in a vicious interaction with under-regulated retail medicines markets. Asymmetric information in these markets creates perverse incentives to sell inappropriate and poor quality medicines. Branding is also used to segment the market and support monopoly pricing for those able to pay [1,7]. A UN medicines expert interviewed for this project argued:

'at every step of the supply chain there is this unequal knowledge, and people are exploited because of that lack of knowledge.'

In India and Sub-Saharan Africa, public purchasing can improve quality and reduce prices, but public funding of drugs is grossly inadequate and often poorly spent [8-11].

International and national, faith-based and secular NGOs have responded by providing and funding health care in both India and Sub-Saharan Africa (SSA), and by campaigning. The recent huge increase in aid funding for medicines for HIV/AIDS, TB and malaria has been routed increasingly through nongovernmental organisations [12]. NGOs including Health Action International (HAI) and the Treatment Action Campaign based in South Africa have campaigned to force down prices of HIV/AIDS and other medicines [13,14]. Furthermore, NGOs have worked with the WHO to develop essential medicines lists that identify the most cost-effective, mainly generic medicines for each major illness. In India, the All India Drug Action Network (AIDAN) [15] of NGOs working to increase access and improve the rational use of medicines has influenced policy, for example by weeding out harmful and irrational formulations.

However the research literature has largely ignored the important role of NGOs in quality assurance and trading essential medicines. Web searches of the medical and social science literature using key terms including drugs, medicines, NGOs, non-profit, wholesaling and trading produced no survey of this activity.

## Theory and methods

### Trading NGOs and market failure

NGO wholesalers of essential medicines can influence access in two ways: by directly improving price, quality and accessibility for users of their products, and also by influencing the behaviour of other market participants. We examine both roles in this article. Economic theory and empirical work has generally focused on explaining the first role. Trading NGOs (for example, non-profit hospitals) are argued to arise in health care as solutions to market failures. The classic statement of this argument is by Kenneth Arrow:

'I propose here the view that, when the market fails to achieve an optimal state, society will, to some extent at least, recognise the gap, and non-market social institutions will arise attempting to bridge it.'[16]

Non-profit firms are argued to have a market advantage because they cannot distribute financial surpluses to shareholders. Hence, they have less monetary incentive than private firms to cheat poorly informed customers by reducing quality in order to increase profits. Customers therefore regard them as more trustworthy and are willing to pay a premium price for more reliable quality [17]. Trustworthiness is further strengthened if people more inclined to ethical behaviour are disproportionately attracted to work in socially oriented firms (a 'selection effect') [18,19].

### NGOs as market regulators?

Much less analytical attention has been paid to the second role [20,21]. However there is accumulating evidence from Asia and the USA that a substantial presence of non-commercial providers in health care markets can influence positively the *quality *of commercial provision [22,20]. An effect of this kind, sometimes called 'beneficial competition' [23], whereby socially oriented enterprises influence the behaviour of commercial firms in the same market, can be characterised as a market *regulatory *effect, since it shapes incentive structures and market outcomes.

That characterisation stems from the socio-legal literature on regulation, which contrasts narrow definitions of formal governmental regulation with broader concepts that include non-state actors [24]. *Formal *regulation is the state's standard-setting, rule-setting and enforcement role, including registration, licensing, inspection of facilities and firms, and proscription of activities such as sale of listed medicines without prescription.

A contrasting *informal *concept of regulation describes the shaping of market behaviour by 'regulatory webs' of actors and discourse [25]. The state is one actor in such webs. Informal regulation can be understood as a discursively produced informal governance structure for a market. Informal regulatory norms are not simply firms' behavioural regularities - though these constitute evidence for such norms - but rather something akin to a 'script' rooted in past experience of expectations fulfilled and in a shared discourse concerning market behaviour [23,26-28]. In pharmaceutical markets, corporate culture may be more influential than state rule-making in shaping risk and outcomes [25].

### Social enterprise

The concept of social enterprise used in this article is more inclusive than the category of firms legally constituted as non-profit [29,30], since we aim to capture cultural and behavioural aspects of the firms studied. Social enterprises are defined as organisations reflecting an 'entrepreneurial spirit focused on social aims' [31,32], or more simply, firms with social aims operating in markets [33].

### Research methods

As part of a broader study of the supply chain of essential medicines from manufacturers in India, Kenya, Tanzania and elsewhere to rural areas in Tanzania, we interviewed social enterprise wholesalers in India, Tanzania and Europe.

Between one and three senior procurement managers were interviewed in late 2006 and 2007 in Europe-based non-governmental actors in the wholesale market for essential medicines for low income countries. We aimed for an exhaustive set of interviews with all important market actors. Given the lack of a pre-existing sampling frame, the organisations were located through web searches for medicines procurement agents and wholesalers, and the list was then snowballed by asking each firm about their main competitors, the key funding bodies, and the main intergovernmental organisations influencing the market. Only one non-profit trader and one relatively small private firm refused to be interviewed. All but one of the UN bodies and charities interviewed procured medicines not only for their own projects but also for sale to NGOs and government sectors in developing countries. We included the non-profit trading arms of charities and inter-governmental bodies, and also interviewed large funders and the WHO (Table 1; 25 interviews in total). The broader project also included interviews with international and Indian NGO activists [14].

**Table 1:** Organisations based in Europe interviewed, by category

Type of organisation	Number of organisations
Non-profit wholesaler	2
For-profit wholesaler	3
Charity wholesaling medicines	2
UN body wholesaling medicines	2
UN body with a regulatory role	1
Other international body purchasing or funding medicines	1
Other international NGO distributing medicines or campaigning	2

In India, NGOs run healthcare facilities such as hospitals and clinics, providing free or subsidized medicines. However a search for NGO wholesale enterprises aiming to influence the supply chain from manufacturers to users showed there is little such activity. Two exceptions were identified and studied: LOCOST (Low Cost Standard Therapeutics) and Community Development Medicinal Unit (CDMU). LOCOST manufactures drugs for sale to other NGOs, and CDMU is a wholesaler distributing medicines to other NGOs. Interviews and data collection with CDMU and LOCOST in 2006-7 were followed by interviews with 17 member organisations of CDMU, and by email correspondence with LOCOST. Except where stated, all data and documents were obtained directly from CDMU and LOCOST.

In Tanzania, the only two NGO wholesalers were interviewed as part of a larger set of interviews and data collection in late 2006 described in detail elsewhere [6]. Six private importer-wholesalers agreed to be interviewed, from a list of ten key firms provided by the regulatory authorities. Senior public and NGO officials were also interviewed. Medicines retailers and non-governmental health facilities were interviewed in four rural districts, and a set of 31 tracer medicines were used for price data collection [6]. Ethical clearance for the study was obtained from a UK university and from the Tanzanian authorities. Written consent forms were used. Interviewees were promised anonymity, and where specific organisations are cited in this article, permission has been sought from interviewees.

Interviews in the three sites were semi-structured. Indian interviews and Tanzanian interviews with private firms were recorded in notes after the interview. All European interviews and the NGO interviews in Tanzania were taped and transcribed. Limited associated documentation was located: published accounts and firms' websites where available, and official reports and business periodical literature, some cited here. Market price surveys in India are used in our analysis of NGOs' impact, as are our own price survey data in Tanzania.

Our interviews therefore contribute to the health literature a unique qualitative data set on NGOs and medicines wholesaling. The evidence is single-round, not longitudinal, and our Tanzanian price data are not drawn from a national random sample. Our evidence of benefits of NGO wholesaling is thus largely qualitative, drawn from interviews with NGO facilities purchasing from the wholesalers in Tanzania and India. Such qualitative evidence is widely used in socio-legal analysis of market behaviour and regulation [24]. It does not permit statistical generalisation.

Analysis for this article cross-referenced ownership structure with aspects of reported business behaviour, and triangulated interviewees' statements about the behaviour of competitors and the evolution of market competition. The article is thus interpretative and exploratory. We set out evidence from the interviews on the business strategies and market contexts that permit social enterprise to exercise beneficial influence on the terms of trading within medicines markets for low income consumers.

## Results and discussion

We combine results with discussion in order to link evidence and interpretation on each point in this mainly qualitative analysis. All evidence cited, including the initial contextual outline of the three markets, is drawn from the interview data unless otherwise stated and referenced. After briefly outlining the three contexts, we first show that quality control is seen by the firms as key to NGO wholesaling success in each market. We then analyse, for the European context, the evidence that NGOs can exert informal regulatory influence on their market. Next, drawing on Indian experience, we show how, in contrast, NGO success can elicit commercial responses that undermine their market position. Finally, we explore the implications of commercial and regulatory changes at the global level and show that there are opportunities opening up for African social enterprise wholesaling in essential medicines to benefit African populations.

### Trading in essential medicines for low income consumers: NGOs in three contexts

In the international market for essential medicines for low-income Africa, trading organisations with a social mission, based in Europe, have played an important but poorly documented role since the 1970s. The firms interviewed stated that they buy predominantly from Indian manufacturers. The market they supply is funded by a mix of developing country government and international donor funding - including the Global Fund for HIV/AIDS, TB and Malaria (henceforth 'the Global Fund') and PEPFAR (the US President's Emergency Plan for AIDS Relief) - alongside substantial out-of-pocket spending by consumers in developing countries.

The wholesalers interviewed sell to government buying agencies and semi-autonomous Central Medical Stores; to international emergency relief agencies and charities such as the International Committee of the Red Cross (ICRC) and *Médicins sans Frontières*, and UN bodies. They also sell to non-governmental organisations, including church-supported buying agencies and charities supplying mainly faith-based and secular NGO facilities [34]. The firms thus supply a 'social' market, supplying government and non-profit sectors. This operates alongside an international private market for medicines for African countries, regulated only by African government import and registration requirements [35], and at the time of the interviews largely unaffected by major funding initiatives.

It is difficult to estimate the size of this social market. In 2006, 33 African countries in the least developed country category were estimated to import medicines worth in total around US$1.6 billion [35]. This figure includes private market imports, but conversely substantial amounts of aid-funded medicines (including emergency aid) escape inclusion in import totals. Proportions of all imported medicines that are procured by governments or NGOs in African countries vary widely and are poorly documented. In Tanzania, local procurement experts estimated that around 70% of medicines consumed were imported in 2006, and about 50% of the market was supplied by government or NGO procurement. By contrast in Nigeria the largely unregulated private medicines market is very dominant [1]. Estimated procurement of medicines for Africa in 2006 (not including vaccines) by seven of the wholesalers interviewed for this project totalled around US$300 million. One major charity refused however to give a figure. This total certainly underestimates total 'social' medicines procurement for Africa.

The International Dispensary Association (IDA) played a pioneering role from 1972 onwards in shaping this market through non-profit wholesaling. IDA was established in the Netherlands with the involvement of student campaigners for essential medicines lists and the rational use of medicines. It aimed to supply reliable generic essential medicines, and it became the most successful of several non-profit traders established in that era, including Christian charities supplying medicines for mission facilities in Africa, some of which have survived. Another large non-profit trader was later spun off from a government department, and medicine procurement and trading arms were established in Europe by two UN agencies.

By the early 1980s for-profit competitors had joined this market, mainly family-owned and entrepreneurial businesses. All those interviewed also supplied entirely or mainly non-profit buyers. One wholesaler's 2006 turnover, for example, was divided roughly 60% sales to government purchasers, 20% NGO buyers including small and large mission customers in Africa and big international NGOs; 15% United Nations; 5% other. This balance varied between firms and over time; major emergencies for example changed the balance of sales.

Within India, CDMU and LOCOST each stated that they sought to address the huge unmet need for access to safe, rationally prescribed medicines. CDMU was set up in Kolkata in 1984 as a Central Drug Marketing Unit of the West Bengal Voluntary Health Association, and became an autonomous organization in 1986. Its goals include [36]: provision of quality essential drugs to member-partners at affordable cost; provision of unbiased information on rational drug use to health professionals and consumers; and negotiating with the government to formulate people-oriented drug policies and weed out irrational and hazardous drugs from the Indian market.

CDMU was perhaps the first organization in India to apply WHO concepts of essential medicines to influence proper use of drugs. This was remarkable in the mid-1980s: the pooled procurement by the Tamil Nadu Medical Services Corporation (TNMSC) and Delhi hospitals have used similar selection exercises only since the mid-1990s [8,9]. CDMU procures medicines for sale only to non-profit member organizations (MOs): NGOs and faith-based organizations providing free or subsidized healthcare. Some purchase drugs regularly, others occasionally, and some only for relief work during natural calamities.

LOCOST was set up in Vadodara (Gujarat) in 1983 and started drug supply operations in 1985. It similarly caters mainly to voluntary health care organizations. Unlike CDMU, which is concentrated in West Bengal, LOCOST products are supplied more widely, through depots in Bangalore and Guwahati to cater to South Indian organisations and to those in the North East.

LOCOST was set up by a small group of health professional members of Medico Friends Circle, an all-India organization of individuals concerned particularly about the rural health situation. They saw that good quality drugs were generally costly; cheaper drugs were not of proper quality; and many essential drugs were not available particularly in remote areas. Initially LOCOST procured drugs from small scale manufacturers. Soon, it began manufacturing on loan licence, i.e. drugs were manufactured for the LOCOST label under LOCOST supervision. LOCOST set up its own small scale manufacturing plant in 1993 to have better control over supplies and quality. It produces over 60 essential medicines in more than 80 formulations (liquid, capsule, tablet) conforming to WHO quality standards, and now manufacturers most of its drugs supplied. Like CDMU, LOCOST has been involved in education, campaigning and advocacy on rational use of medicines, safety, and pricing and it is an active member of AIDAN.

In East Africa, NGO faith-based wholesalers are well established in Kenya and Uganda. In Tanzania the government wholesaler supplies around 50% of the local market, while a faith-based wholesale presence, small but expanding in 2006, aims to complement it by filling in gaps in supply. Action Medeor Tanzania, a non-profit wholesaler with German support, was supplying local NGO facilities; Mission for Essential Medical Supplies (MEMS), a donor-supported local NGO, brokered and supported effective purchasing by church-owned facilities. In four rural districts studied, most NGO hospitals, but fewer than half of NGO dispensaries and health centres, purchased medicines from the government or one of the NGO wholesale suppliers; the others bought medicines on the private market [6].

### Quality assurance at low prices: the key value-added

All the European firms interviewed, when asked about their value-added, cited quality assurance and quality control of low priced, mainly Indian-sourced medicines. The IDA, the largest independent non-profit wholesaler, said that it addressed this aim by supplying mainly its own-brand generics: 80% sourced in India to reduce prices, pre-packaged by manufacturers with IDA labels. IDA quality assurance and quality control included approving manufacturing sites for each product, and testing all batches; a manager stated:

*Our logistics buyer..... told me.... if the doctors would see that they are getting IDA products, they would be happy ... for them it's really trust and guarantee of quality*.

In 2006, IDA still tested batches in the Netherlands: an expensive process increasingly constrained by EU regulations. Only one other (for-profit) firm branded some of their bought-in generic medicines and also tested all batches en route to Europe. Some competitors disagreed with batch testing as the best route to ensuring quality, and most regarded it as financially unviable, as a for-profit firm's manager commented:

We do not re-analyse all batches, because then we would certainly be non-profit!

The European essential medicines wholesalers were, they stated, under increasing competitive pressure, and the interviews included mutual accusations of resultant weakening of quality assurance. Quality assurance requires close knowledge of suppliers and attention to documentation. Of the five independent wholesalers interviewed, two non-profit and one for-profit firm did their own repeated inspections of manufacturing sites. One used only suppliers they had approved themselves. At the time of the research, the WHO had recently begun 'prequalification' inspections of production of anti-retroviral medicines [37], and these were accepted by some wholesalers. One UN purchasing body and an international charity did their own inspections or contracted for them. The other UN body, the other international charity and one for-profit firm did no inspections, either buying only from European sources (at higher prices) or using as procurement agent another organisation that in turn did the quality assurance.

In India, both LOCOST and CDMU successfully undercut commercial market prices, but only LOCOST had ensured robust quality control. CDMU had undercut high-margin retail prices for MOs that were too small to float tenders, and had ensured supplies even in remote areas. CDMU prices were compared to commercial retail prices for 18 large selling products using Indian market survey databases [38,39]. CDMU prices were found to be lower for 17 out of 18. Retail prices exceeded CDMU price by between 1721.5% (nimesulide) and 83.3% (ampicillin/cloxacillin)[2].

LOCOST similarly improved affordability of medicines [40] (Table 2)

**Table 2:** Comparison of LOCOST and market prices, selected medicines

Drug	LOCOST price	Market price
Albendazole	Rs. 11.0 per 10 tabs	Rs 9- Rs.12 per tablet
Amlodipine	Rs. 2.50 per 10 tab	Rs. 14 to Rs. 48 per 10 tabs
Atenolol 50 mg	Rs. 2.80 per 14 tab	Rs. 4- Rs. 22 per 10 tab
Enalapril 5 mg	Rs. 3.0 per 10 tabs	Rs. 16- Rs. 23 per 10 tabs
Fluconazole 150 mg	Rs. 35.00 per 10 tabs	Rs. 28-32 per 1 tab

Source: [40]

The challenge, as for all the firms interviewed, was to combine lower prices and quality control with financial stability. CDMU has consistently struggled financially. Initially it grew fast: MOs registered rose from 38 in 1986 to 396 in 1997-98, and sales from Rs 2.23 million in 1986 to Rs. 18.4 million in 1997-98. Since then however sales have fluctuated but stagnated, while CDMU has incurred losses almost every year since 1986, funding those losses though donations.

The main reason is CDMU's persistent weakness in quality assurance. Among large MOs that dominate CDMU procurement, Howrah South Point, for example, installed testing equipment and found sub-standard drugs; a problem CDMU failed to rectify. Two others, Antara and Calcutta Rescue, reduced their purchases for similar reasons. CDMU has from time to time adopted basic physical testing in-house and analytical testing by external government approved laboratories. Some manufacturers have been black listed. However, CDMU never achieved effective quality assurance.

As a result CDMU's Kolkata office incurred persistent losses since it could not retain major purchasers. In 2002-03, 77% of sales were to just 18 MOs, each with procurement above Rs 100,000; by 2007-8 the share of these 18 had declined to 43%, and 4 had left CDMU. Only CDMU's Branch Office Siliguri, handling 40% of total sales, made a financial surplus. Small scale procurement by tea gardens that run health facilities in remote areas of North Bengal accounted for 94.5% of total Siliguri sales; these buyers have few other procurement options.

In contrast to CDMU, LOCOST generates a surplus. Its drug sales doubled between 2000-01 and 2007-08 to Rs 25.47 million. LOCOST has an in-house quality-control laboratory where medicines are tested before being supplied. Even when some drugs are available at lower prices in the market, some NGOs continue to buy from LOCOST because of the quality assurance. LOCOST officials argue that they respond seriously to quality complaints and have earned most customers' trust. The organisation's financial surplus has funded minor plant expansions, and it has gained Ford Foundation and Bread for the World grants between 2001-5 to fund upgrading to meet revised Indian government regulatory requirements based on WHO Good Manufacturing Practice (GMP) guidelines. It has however stopped manufacturing liquids because it could not afford the upgrading costs.

LOCOST has been the more successful at quality assurance in good part because it appears to function with a stronger sense of values and purpose than CDMU. One of LOCOST's founders, S. Srinivasan, was its Managing Trustee and continued to guide its strategy. The management structure was clear; the two managers were well qualified and quite long-serving; and the staff worked flexibly. CDMU in contrast had failed to create an effective and value-based management structure. It was run by an Executive Committee without a strong administrative head with proper autonomy and accountability. Lack of proper management coordination and the inability to take prompt actions in Kolkata had left problems unaddressed, including complaints of uncooperative and unresponsive behaviour by some CDMU staff.

The two Tanzanian NGOs took different approaches to quality assurance. MEMS in 2006 was assisting faith-based hospitals to upgrade their stock control and ordering. Their orders went through a local private wholesaler who ordered imports from IDA and relied on IDA quality assurance. MEMS also did some quality control checks using mini-labs and local laboratories. MEMS was at the time 90% donor-funded, and also charged a commission on sales.

Action Medeor Tanzania had a warehouse in Dar es Salaam; the initial investment was made by Action Medeor Germany in 2004. This Tanzanian NGO procured around 60% of their medicines from Tanzanian and Kenyan suppliers, and did its own regular plant inspections. They also inspected all batches and did random testing using a WHO-prequalified laboratory in Kenya and Tanzania Food and Drug Authority (TFDA) facilities. The other 40% came from European manufacturers, for example in Cyprus, or from India via IDA relying on IDA quality assurance. When interviewed, the firm was working towards covering costs from their mark-up.

Both Tanzanian NGO wholesalers bought efficiently, undercutting commercial wholesalers. For 24 tracer medicines that were bought by all wholesalers interviewed, the NGOs (like the public wholesaler) were buying at significantly lower prices than the private wholesalers in 2006, and passing on these savings in lower prices to NGO facilities as compared to private sector facilities' buying prices [6].

### Shaping a social market: NGO benchmarking in the European market

Given the market incentives to reduce quality, what mechanisms keep many NGOs' behaviour focused on providing good quality, thus sustaining merited trust from buyers? And to what extent does NGO presence influence the company culture of competing firms. The literature on NGO health services in Africa and in the USA attributes trustworthiness mainly to religious values-driven commitment to patients [41,42]. However the cultural values of the Europe-based international traders had their roots in a more diverse mix of left wing political engagement, religious mission-linked commitment, and public sector procurement agency experience.

The for-profit European firms interviewed all claimed a social mission that resembled that of the non-profit traders: for example, one expressed it as '*expanding the availability of generic pharmaceuticals worldwide*'. Several had their origins in the non-profit sector. One early charitable trader had by 2007 been taken over by a commercial firm. The procurement manager explained the history:

*When they came back [from mission work in Africa] the owner and his wife started the business in their garage. It was a pure charity*.

The new commercial owner had retained a nucleus of experienced and committed staff from the charity, and had also segregated the activity physically away from the '*purely commercial' *culture of the rest of the firm, in a unit with its own culture and management.

Another for-profit business had been started by a founder of one of the non-profits. A third commercial firm's founder had taken the African wholesaling business out from a commercial wholesaler and established it independently as a family business. Asked why this business model was chosen, the general manager said:

*He ended up doing it as a private company because that was easier than to make it a foundation *[that is, a non-profit enterprise]'

Furthermore, the stated 'social mission' of the for-profit firms is a tool of effective competition in this market. All these firms stated that it attracts socially motivated staff and constitutes a signal of commitment to good quality. Each firm, or separate division, mainly or solely supplied non-profit, inter-governmental and government buyers. All emphasised that this was a market with rather few major players, so reputation was key: several firms said their 'core business' was repeat orders based in long term working relationships.

We asked each organisation whether non-profit status in itself now constituted a market advantage, and the predominant view was that it did not. The for-profit firms were eligible to bid for most business, and while they also sold to private buyers, each said it was a very small part of their business. The non-profit wholesalers did not sell to the private sector.

The experience of the charity that became a division of a commercial firm illustrates this point, as the procurement manager explained:

*We thought initially the change from a charity to commercial might have a negative impact, and it wasn't, after the first three months - most customers came back*.

The firm lost charitable discounts from suppliers - of UK equipment in particular - when it ceased to be a charity, but said suppliers observed them still working in the charitable market, saw that '*the customers are still the same' *and that price lists showed no big mark-ups, so '*they are coming round*'. The specialist focus on the 'social' market was presented as implicit evidence of lack of profiteering, alongside the explicit social mission. The marketing manager of the larger commercial firm owning this division emphasised that he had had to learn a different, less commercially aggressive marketing style for this part of the business.

This 'social market' is thus a strongly relational market: one interviewee called it '*personalised*', requiring '*constant talking to customers and suppliers*'. Some interviewees had spent their working lives in this market, and knew their competitors well ('*the usual suspects'*). These interactions have in turn shaped the informal regulatory influence of non-profit enterprise, since the cultural and behavioural feedback between firms is very direct, allowing the weight of the non-profit traders to influence the strategy of commercial firms in the direction of social enterprise behaviour.

The benchmarking influence of one major firm, the IDA, on the market's regulatory norms emerges particularly sharply. Analysis of the interviews showed that in interviews with every competitor and with most international organisations, the IDA was mentioned unprompted. Aspects of firms' strategy were explained with reference to the IDA. Thus one charity began to explain their niche by saying: '*we are not sort of, we are not an IDA'*, meaning not a non-profit wholesaler nor very large within the market.

When asked what difference non-profit as opposed to commercial status made in this market, several other firms defined themselves in relation to the IDA. For example, on product range:

*we tend to be quite flexible in the range of articles we supply, which is not similar to what IDA does in maintaining a fixed list of essential drugs which they claim to be very good value, in some cases they are *(for-profit firm)

And on prices, a charity said:

The thing is our prices are, compared to other organisations like IDA ...relatively high.

Two for-profit players said unprompted that the relationship with the IDA had shaped their mission and strategy. As one put it:

*we share a lot of history, you know, in the beginning back from '75 to '78 we, you know, there was a very close co-operation between IDA and [ourselves] *(for-profit firm)

In this case the relationship had later become more competitive. Two for-profit companies had cooperated in buying for a while, in order to get the volumes that would allow them to compete:

*because the big, big company in the business was IDA*.

One for-profit firm argued - slightly tongue-in-cheek - that as far as:

*the commercial aggressive approach is concerned I would say eh, for many years IDA has been by far the most aggressive player in the business*.

This evidence of IDA's key role in the discursive and practical construction of market behaviour shows IDA acting as a market-maker - being the first big independent player - and as a benchmark firm and beneficial competitor in the market as it evolved. According to our interviewees it has influenced culture and helped to keep down prices and put a floor under quality by providing a 'fall back' with known prices and reliable quality. This benchmark role has also influenced the expectations of downstream buyers. A charitable trading company manager confirmed this, arguing furthermore that their own role in the market had also influenced the behaviour of the commercial firms, notably on quality:

*our wholesalers are used to our high quality expectations, so I think in a way we triggered the market, although we are the minor player .....And the same goes for IDA*.

The interaction of NGOs' behaviour and buyer expectations had thus shaped a Europe-based social market supplied by enterprises - non-profit and for-profit - with a distinctive social enterprise culture and terms of trading.

### Commercial responses and pressures on NGO traders

Medicines manufacturers, however, are affected by NGO trading, and the trading practices of social enterprises create new market opportunities that invite commercial response. NGOs in all three sites have been affected by the commercial responses of Indian manufacturers.

CDMU's experience illustrates this type of problem. CDMU's intervention in the Indian medicines market, coupled with changes in the industry, altered relationships between MOs, manufacturers and distributors. CDMU's tender system is transparent: its Price List issued to MOs specified the names of manufacturers. This information then allowed larger MOs to approach the manufacturers and negotiate directly. CDMU levies a service charge of 10% on the drugs supplied, so directly approaching manufacturers is cheaper for large MOs. Moreover, CDMU's success in expanding sales in the early years attracted the notice of some manufacturers, who could obtain the names of the MOs from the loosely structured administration of CDMU. Some manufacturers/distributors then approached the larger MOs, profiting by avoiding tendering costs and hassle. A financially unstable CDMU could not always pay the suppliers on time, so direct supply to MOs meant prompt payment. In such cases they could even undercut the CDMU tender price. Many manufacturers now supply large MOs directly.

CDMU also effectively introduced distributors to MOs. Over time, these distributors started supplying other drugs, and became competitors of CDMU. Thanks to CDMU, MOs now know the market much better, and now shop for themselves, even using tenders. Some MOs have found drugs available in the wholesale market at prices lower than CDMU prices (including metronidazole, mebendazole, ranitidine, cotrimoxazole, ciprofloxacin). If CDMU guaranteed quality, then some MOs may have preferred CDMU despite higher prices. In its absence CDMU loses markets.

LOCOST is not immune to these pressures. Despite its successes, expanding sales has not been easy and it remains a relatively small market player: out of 468 companies in the retail formulations market in India listed in the market surveys by ORG-IMS [43], 271 had retail sales greater than LOCOST's in 2007-08. LOCOST's competitors furthermore began to take note of it as it grew. The pharmaceutical market has become very competitive, and the recent upgrading increased operating expenses and removed some of LOCOST's competitiveness. Pharmaceutical companies' active marketing includes incentives and inducements to influence doctors, consumers and drug procuring institutions. However LOCOST spends nothing on marketing. This keeps its costs and prices low, but has also put it at a competitive disadvantage when dealing with organizations that are susceptible to marketing gimmicks and incentives. LOCOST - like CDMU - has also lost customers because of its policy of restricting its sales to rational formulations.

In the European market too, Indian manufacturers try to undercut the role of social enterprise. This social market has patchy market information, and national governments' buying and handling capability is uneven. There are many conflict and emergency situations, and here too the independent wholesalers have been market-makers. The interviews with wholesalers show that they link quality assurance to assemblage and logistics, strengthen supply chains, and complement direct procurement by big international charities and United Nations bodies. They can assemble complete parcels or kits, rapidly and at high volume, from different manufacturers. The main firms stock large warehouses - for example, IDA could supply 750 items from stock in 2006 - tying up substantial working capital. One interviewee estimated US$5 million in stock was required to be an effective wholesale market player.

However, market strategies of the Indian pharmaceutical companies threaten the viability of these activities, and by 2006-7 were forcing a move of wholesaling out of Europe. Since the mid- to late-1990s, Indian manufacturers have increasingly supplied some large buyers such as government Central Medical Stores directly, by-passing the European wholesalers. This created intense price competition for large tenders, squeezing wholesale margins, and all the firms were stated that they were struggling to sustain quality assurance while drastically lowering costs.

The main European-based wholesalers were therefore, when interviewed, in the process of moving much of their warehousing and logistics to India in an effort to cut costs. The move was also driven by increasing stringency of regulations concerning import of medicines into the EU. The move was difficult, not least in dealing with the complexity of legal and tax rules for foreign companies operating in Free Economic Zones in India; at least one firm, according to interviews and annual reports, was losing money during the process.

Competition from manufacturers' direct sales was said to be particularly strong where purchasers were large, efficient and well informed. One European wholesaler described a learning process parallel to the Indian NGOs' experience:

*What we were mainly doing is telling these guys where it *[the product] *is coming from, so we are educating our customers*

The manufacturers also benefited from wholesalers' investment in market-making when wholesalers register a manufacturer's product in an African market, and establish the product's reputation:

*And then you know, when everything is registered, which takes a long time ... costs you a lot of money ... then they start selling directly. ... we make the market and then they come in and take over. *(for-profit firm)

Two of the for-profit wholesalers interviewed were diversifying into manufacturing, through joint ventures with Indian firms, in order to learn about manufacturing and to increase control and flexibility in supplying customers. Wholesalers retained their added value when assembling large lots of diverse medicines for emergency supplies and kits for primary health facilities, and when responding to large complex tenders for Central Medical Stores which might require contracts with dozens of manufacturers if purchased directly. But increasingly warehousing and logistics had to be done in India to stay competitive.

Another competitive tactic was to go more into supply chain management within countries; as one manager explained:

We are very good in the post-war countries or in the countries where there is ... disorganisation. When the country is getting more mature, then we are losing market share.

For example, one for-profit firm was undertaking a complex project that required support for local manufacturing firms in a conflict-ridden country, including raw materials supply to manufacturers and local assemblage and delivery of local and imported supplies. One large non-profit trader had long supported procurement capacity development, including training, in developing countries.

The growing market for supply chain management was also driven by major new funders. The Global Fund, PEPFAR and the Global TB Drug Facility have financed high volumes of pharmaceuticals for HIV/AIDS, malaria and TB. They typically require procurement agents to buy and organise delivery on behalf of the recipients of the funds. The huge sums flowing through these market channels after 2004 forced existing wholesalers to rethink their roles, and the volumes on offer attracted large firms as new market players. PEPFAR's Supply Chain Management System (SCMS) brought in some US-based firms. The UNDP set up IAPSO, its procurement arm, in 2004, to support in-country procurement using Global Fund resources.

Established wholesalers then had to choose, as a for-profit company director explained, between competing for a major role as procurement agent for the big funds, or being sidelined as a minor market player. A specialised arm of IDA (then called IDA Solutions) was doing antiretroviral (ARV) procurement for PEPFAR. Increased concentration of buying power and procurement had created closer market relationships, with a few intermediaries playing multiple roles in bidding, issuing tenders, wholesaling, and acting as purchasing agents: '*corporatism'*, one UN procurement manager called the emerging market structure.

The firms interviewed also served areas of the market - such as supplies to the UN and some big international charities - that were less price sensitive, with less tendering and more emphasis on speed, reliable response, and safety as represented for example by supply of UK-licensed generics (which, one interviewee said, have become more competitive as Indian firms have bought UK manufacturing plant and licences):

*We may be slightly more expensive but we tailor to their needs *(for-profit firm)

A large international charity confirmed that they did not generally issue tenders, relying on repeat orders with established suppliers.

Many interviewees argued however that there had been an over-emphasis on driving down prices of standard items, such as basic antibiotics sourced in India, through tendering:

*For many of those products we are down to rock bottom prices and there is actually exit from the manufacturers who produce them *(UN interviewee)

Experienced wholesale buyers felt increasingly trapped between pressures that worsened market incentives to cheat:

*you can't have wildly diverging things ... someone saying, oh, you've got to get the prices down, and by the way you've got to have this quality standard, the golden standard ... they will try to cut corners *(UN interviewee)

The interviews included a number of anecdotes about tenders accepted on price alone producing poor quality. Repeated worries were expressed that independent wholesaling was being squeezed out and the market '*skewed'*, weakening broader medicines procurement.

Procurement managers interviewed were also finding the number of reputable suppliers becoming dangerously small, as Indian manufacturers turned to more profitable use of their production lines. The most reputable Indian manufacturers were losing interest in supplying basic generics to the low priced African market except in key high volume areas such as ARVs, and the Tanzanian market was therefore increasingly supplied mainly by second tier Indian firms with less strong quality reputations [35].

### Social enterprise wholesaling in Africa: a developmental opportunity?

The commercial responses to social enterprise trading in essential medicines have opened up opportunities for a developmental role for Africa-based social enterprise. The two Tanzanian NGO traders interviewed had strong European NGO links: both purchased from IDA, and one was a 'daughter' company of a European charitable trader. Furthermore, like the Europeans, the Tanzanian firms were selling into a strongly defined social market segment of Tanzanian health care, the faith-based and NGO facilities, and interacting with their culture and values. This market segment was also strongly influenced by the large public wholesaler which supplied many NGO facilities and exerted downward pressure on prices. The NGO wholesalers did not supply the private sector.

Tanzania continues to require trustworthy quality assurance intermediaries between manufacturers and buyers. The WHO pre-qualification of medicines for AIDS, TB and malaria focuses on medicines for which there is high market concentration among buyers and suppliers, and works well where the costs to a supplier of being caught cheating on quality or source of supply are high. In the wider essential medicines market, the incentive structures remain problematic, since high numbers of plants supplying a large range of medicines cannot be constantly re-inspected, and wholesalers are therefore needed to assemble large orders, check origins, and guarantee quality and the integrity of the whole supply chain.

The European firms were encountering no international competition from Indian social enterprise in wholesaling. None of our interviewees could identify an Indian social wholesaler in the export of essential medicines from India to Africa; one commented that this was odd, given Indian entrepreneurial flair. Reasons suggested included the relatively undeveloped nature of social entrepreneurship in India and its concentration on its domestic market problems, along with a lack of concern in India with African problems.

It follows that a space has opened up for African and Africa-based social entrepreneurship, as the two Tanzanian NGOs interviewed made clear. Action Medeor Europe said that it had specifically chosen to move, not to India but to Africa:

*we had actually the choice between opening something in Asia, like IDA ... to reduce cost ... or an alternative way could have been to open a local *[African] *office where you buy strictly locally registered drugs..... And we thought, which would be more helpful for the country?*

The charity therefore decided to set up a non-profit wholesaler in Tanzania, to sell to the non-profit sector locally and perhaps to export to the region. A strength of this experiment is that it has clear synergies with the interests of the growing local pharmaceutical manufacturing industry in East Africa, including Tanzania. Shut out of the Global Fund 'segment' of the market to date by the stringent requirements of WHO prequalification, the local industry has been successfully upgrading and needs markets to develop [35]. A shift of social enterprise wholesaling towards supporting this growth - by undertaking their own quality assurance - has the potential to support this development and provide an alternative location for social enterprise regulatory impact.

## Conclusions

This study has documented a largely unstudied segment of essential medicines markets, NGO wholesaling, through interviews with most NGO traders, with NGO facilities buying from those wholesalers, and with for-profit competitors in Europe and Tanzania. The methodology is qualitative and exploratory. The interviews provide a quite exhaustive snapshot of the business history, organisation and behaviour of this NGO sector in India, Europe and Tanzania. The data set is not longitudinal and the international 'social' market in particular still lacks quantitative documentation outside the segment of high-profile large scale procurement of HIV/AIDS, TB and malaria medicines. Our findings strongly argue however for the importance of this sector for promoting access to essential medicines by the poorest people. Furthermore, our findings, as summarised below, can generate hypotheses for future research.

The social enterprise traders studied in this article are all addressing a huge problem: the large numbers of low income people who lack access to safe, rationally prescribed and appropriate medicines in India, Africa and elsewhere. All the enterprises had intervened effectively to improve access. Those that had sustained quality assurance and control, while achieving financial stability including access to support from donors, demonstrated that social enterprise in manufacturing and distribution can effectively create sustainable low cost procurement options for organizations serving the most disadvantaged. Such direct impact on access to medicines depends in turn on effective value-based management and the capability to retain value-oriented staff.

We have also shown that medicines markets and manufacturers respond competitively to NGO intervention, forcing social enterprises repeatedly to rethink their strategy. In particular, transparent procurement by NGOs can improve market functioning but also open up opportunities for direct supply by manufacturers to large organisations that undermine in turn the role of the NGO wholesalers. Social enterprise traders are thus under constant pressure from their own success.

Third, we have shown that social enterprise medicines traders can exercise a broader informal regulatory influence on their markets by influencing the norms and culture of their commercial competitors. Success in this requires not only effective, value-based management and quality assurance capability, but also funding bodies and purchasers with a social commitment to quality at low cost, and NGOs that are large enough relative to the market to play a benchmarking role. In India, the NGOs have not attained that scale except where, as in the 'Delhi model', they work with public procurement. In Europe, IDA and other NGOs effectively played that role from the 1970s past the turn of this century, though the market is now in flux. In Tanzania, there appears to be space opening up for an effective role working with public wholesalers and NGO facilities; the bonus is that in Tanzania as perhaps elsewhere in East Africa, effective social entrepreneurship of this type could help to support local industrial reconstruction and more secure pharmaceutical supply.

The social enterprises studied here were partially substituting for weak formal regulation. Formal regulation has improved somewhat in the international and Tanzanian markets in recent years. However all these markets continue to be weakly regulated, and some recent market pressures have strengthened incentives to reduce quality. It follows that social enterprise wholesaling of the type discussed here needs to be appreciated, sustained and promoted by health policy makers unless or until formal regulation becomes much more effective.

## Competing interests

The authors declare that they have no competing interests.

## Authors' contributions

All authors designed the research. The jointly designed interviews used in this article, and initial analyses of those interviews, were undertaken by: MM in Europe, SC in India, and PGMM, MM and SC in Tanzania. MM led the drafting of this article; SC drafted the Indian results; all authors participated in redrafting and agreed the final manuscript.
